# Reversal of drug-resistance by noscapine chemo-sensitization in docetaxel resistant triple negative breast cancer

**DOI:** 10.1038/s41598-017-15531-1

**Published:** 2017-11-20

**Authors:** Ravi Doddapaneni, Ketan Patel, Nusrat Chowdhury, Mandip Singh

**Affiliations:** 1 College of Pharmacy and Pharmaceutical Sciences, Florida A&M University, Tallahassee, FL 32307 USA; 2 Department of Ophthalmology, Bascom Palmer Eye Institute, University of Miami Miller School of Medicine, Miami, FL 33136 USA; 3 College of Pharmacy and Health Sciences, St. John’s University, Queens, NY 11439 USA

## Abstract

Multidrug resistance (MDR) is a major impediment to cancer treatment. Here, for the first time, we investigated the chemo-sensitizing effect of Noscapine (Nos) at low concentrations in conjunction with docetaxel (DTX) to overcome drug resistance of triple negative breast cancer (TNBC). *In vitro* experiments showed that Nos significantly inhibited proliferation of TNBC wild type (p < 0.01) and drug resistant (p < 0.05) TNBC cells. Nos followed by DTX treatment notably increased the cell viability (~1.3 fold) markedly (p < 0.05) in 3D models compared to conventional 2D systems. *In vivo* oral administration of Nos (100 mg/kg) followed by intravenous DTX (5 mg/kg) liposome treatment revealed regression of xenograft tumors in both wild type (p < 0.001) and drug-resistant (p < 0.05) xenografts. In wild type xenografts, combination of Nos plus DTX group showed 5.49 and 3.25 fold reduction in tumor volume compared to Nos and DTX alone groups, respectively. In drug-resistant xenografts, tumor volume was decreased 2.33 and 1.41 fold in xenografts treated with Nos plus DTX significantly (p < 0.05) compared to Nos and DTX alone respectively and downregulated the expression of anti-apoptotic factors and multidrug resistance proteins. Collectively, chemo-sensitizing effect of Nos followed by DTX regime provide a promising chemotherapeutic strategy and its significant role for the treatment of drug-resistant TNBC.

## Introduction

According to the American Cancer Society, approximately 246,000 new cases and 40,000 deaths in the United States were reported from breast cancer in 2016^1^. Multidrug resistance (MDR) is considered a major impediment to cancer treatment because most cancer-related deaths are due to metastatic tumor resistant to chemotherapy^2–4^. Emergence of drug-resistance often contributes to failure of drugs and poor prognosis, and thus necessitates development of new and improved modalities to treat triple-negative breast cancer (TNBCs)^5^. Moreover, it is increasingly recognized that tumors show high molecular heterogeneity^6^, thus drug resistance can arise through therapy-induced selection of a resistant minor subpopulation of cells that was present in the original tumor. Hence, effective therapeutic modalities are urgently needed to overcome multidrug resistance of cancers and improve outcomes.

Chemo-sensitizers or efflux pump modulators could be one of the possible choices as therapeutic enhancers of chemotherapeutic drugs to decrease their cytotoxicity; therefore, it is important to select chemo-sensitizer agents which are less toxic and more beneficial to the cancer patients. Natural compounds have been explored to act as potent chemo-sensitizers in combination with conventional chemotherapeutic drugs^7,8^. Thus, the identification of chemo-sensitizers that are pharmacologically safe over several synthetic chemicals for their administration with cytotoxic agents in combination therapies against drug-resistant tumors is crucial. Noscapine (Nos) has been extensively investigated as a single agent anticancer therapy against melanoma, lung, prostate, ovarian and breast cancers and it acts through various mechanisms such as binding to microtubules like taxanes, inducing apoptosis and inhibiting angiogenesis^9–14^. Previously published reports including our laboratory findings have provided evidence that enhanced tumor growth inhibition of various tumors was achieved by combining Nos with chemotherapeutic drugs^14–17^. Further, our group also demonstrated that Nos mainly acts through the inactivation of NF-kB and anti-angiogenic pathways while stimulating apoptosis and enhancing the anticancer activity of doxorubicin in a synergistic manner against TNBC tumors^18^. Thus, even though Nos cannot be used as a standalone agent in TNBC treatment, its chemo-sensitizing effect can be critically important for enhancing the tumor specific toxicity of anticancer drugs. Till now, to our knowledge there is no report available for low dose oral Nos therapy as chemo-sensitizing agent for taxanes against TNBC.

Despite these advances, most of these strategies used alone cannot control and maintain the reversal of the MDR phenomena due to the poor tumor-targeting property of these agents in free forms^19,20^. To address this dilemma, nanoparticle-based drug delivery systems have attracted more attention for enhanced MDR reversal in cancer therapy^21,22^, which can efficiently deliver the therapeutic agents to the tumor tissue by the enhanced permeability and retention (EPR) effect^23,24^. The PEGylated liposomes are efficient drug carriers that can evade rapid clearance by the reticuloendothelial system of the body^25,26^. Many liposomal drugs are already approved for clinical use, such as AmBisome, Doxil (Ben Venue Laboratories, Inc Bedford, OH), DaunoXome, Marqibo and Myocet (GP-Pharm, Barcelona, Spain), while others are under clinical trial. Nos chemo-sensitizing effect can be critically important for enhancing the tumor specific toxicity of DTX liposomes and will help in reducing the dose of DTX and its dose dependent side effects. Docetaxel loaded PEGylated liposomes (DTXPL) were prepared and characterized by our group in non-small cell lung tumor bearing mice^27^.

Poor availability of anticancer drug and nanocarrier in solid tumor is one of the major limitations in their therapeutic outcome^27–29^. In such scenario, stromal disruption could be important for harnessing the potential of anticancer therapy. In our previous report, respiratory and oral delivery of telmisartan showed significant anticancer and antifibrotic effects in orthotopic and metastatic lung tumor models^29,30^. Kach *et al*. (2014) demonstrated the anti-fibrotic activity of Nos through cAMP/PKA signaling activation mediated by prostaglandin E2 receptors in pulmonary fibroblasts^31^.

In our laboratory, we demonstrated that low dose Nos acted as a chemo-sensitizer and efficiently inhibits the growth of TNBC cells followed by DTX treatment may produce superior anticancer effects. In our recently published study, we have evidently showed that Nos treatment lead to the activation of early stress markers such as phospho p38 and phospho JNK (family of MAP kinases) in a time and dose dependent manner, thus may sensitize TNBC cells to DTX to induce apoptosis significantly. In the same study, we published that Nos could act as an anti-fibrotic agent and enhance the tumor penetration of coumarin-6 loaded PEGylated liposomes in triple negative breast cancer xenografts^32^.

In this study, we have proposed to treat both wild type and drug-resistant breast tumors with Nos by oral route prior to administering the nanoparticles to solid breast tumors. We hypothesize that prior treatment with Nos will make poorly penetrable fibrous tumors into easily nanoparticle penetrable loose interstitial networks allowing for superior intratumoral distribution of the nanotherapeutics leading to their superior anticancer effects as well as to overcome drug-resistance.

## Results

### Noscapine increases the sensitivity of drug-resistant TNBC cells to DTX

**Figure 1:**
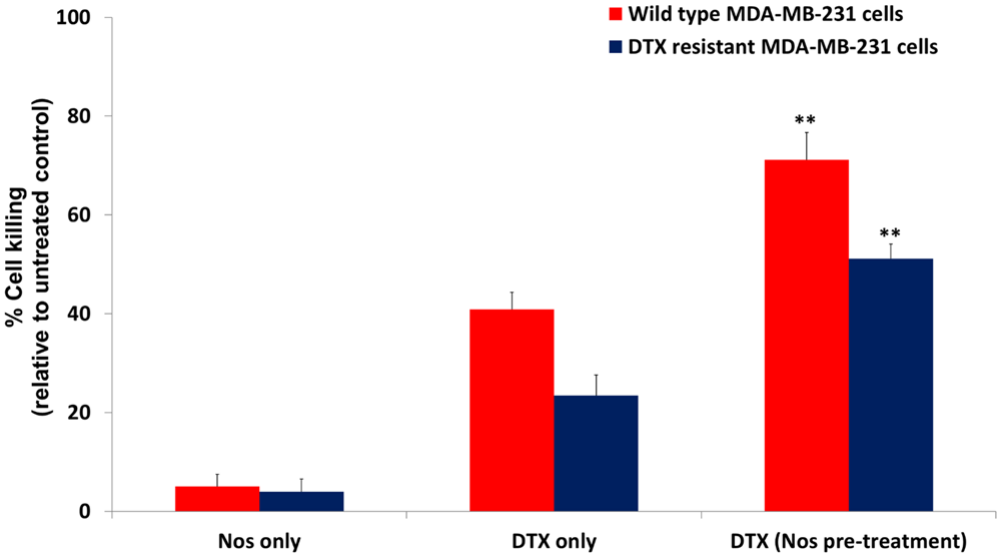
Noscapine pre-sensitization enhances cytotoxicity of wild type and drug-resistant MDA-MB-231 cells followed by docetaxel treatment. Triple negative breast cancer cells were pre-sensitized with noscapine for 24 h followed by docetaxel treatment for 24 h and percentage cell killing was measured by the crystal violet assay. Each value represents the average of the independent experiments with triplicate determinations. *Indicates a significant (**p < 0.01) difference compared with control. Data presented are means ± standard deviation (SD).

Our previous studies have indicated cancer cell growth inhibitory properties of Nos after pre-sensitization and also enhancement of anticancer activity of DTX^32^. In the present study we examined the pre-sensitization effect of Nos at low concentrations in drug-resistant TNBC cells and compared with the wild tumor cells. First, the wild type cells were exposed to Nos for 24 h followed by DTX and their cytotoxicity was determined. To minimize extensive loss of cell death after treatment, a dose of 8 μM Nos and/or 0.8 μM DTX was chosen for a 24 h treatment period. Treatment with Nos alone does not show cytotoxicity of wild type cells but as shown in Fig. 1, cells treated with Nos at low concentrations followed by DTX treatment markedly (p < 0.01) increased the cytotoxicity of wild-type TNBC cells. Cells which were treated with DTX only showed 40.0% cell killing but Nos pre-treatment followed by DTX treatment group showed 74.0% cell killing.

Further, we wanted to investigate the pre-sensitization effect of Nos on drug-resistant TNBC cells. Treatment of drug-resistant TNBC cells with Nos at low concentration followed by DTX resulted in a significant increase in the cytotoxicity when compared to cells treated with DTX alone (p < 0.05, Fig. 1). In DTX resistant TNBC cells, only DTX showed 23.41% of cell killing but Nos plus DTX showed 52.03% cell kill as compared to control. There was no significant difference between the percentage of cytotoxicity in cells treated with Nos alone at low concentration and control cells. We found that Nos chemo-sensitization followed by DTX treatment significantly (p < 0.01) increased cytotoxicity of DTX in resistant MDA-MB-231 cells.

### Noscapine chemo-sensitization suppress three-dimensional growth of the drug-resistant TNBCs

**Figure 2:**
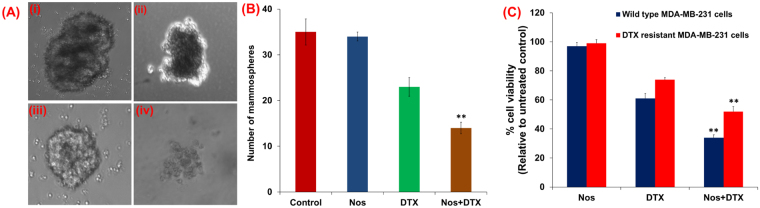
Noscapine pre-sensitization followed by docetaxel treatment inhibits growth of mammospheres derived from drug-resistant TNBC cells. (**A**) Drug-resistant MDA-MB-231 triple negative breast cancer cells were grown as mammospheres as detailed in methods. The mammosphere cultures were either untreated (control) or treated with Noscapine and docetaxel for noted dose and time. The representative microscopic images of mammospheres were showed in Fig. 2A. (**B**) Number of mammospheres of drug-resistant TNBC cells in each treatment group (control, Noscapine alone DTX alone, noscapine presensitization followed by docetaxel) was quantified microscopically. (**C**) The cell viabilities of both wild type and drug-resistant TNBC cells were determined by alamar blue-based assay as shown in Fig. 2C, and plotted relative to the values for the respective untreated controls. The histogram columns in panels 2B and 2 C represent means of three independent experiments, respectively. *Indicates a significant (**p < 0.01) difference compared with control. Scale bar 400x. Error bars represents ± SD.

In order to determine the efficiency of Nos pre-sensitization effect, TNBC cells were grown in 3-dimensional (3D) cultures because this system mimics *in vivo* system. Cell viability of both wild type and drug-resistant TNBC cells in 3D alginate scaffold matrix was shown in Table 1. In our lab we already have optimized the 3D alginate scaffold using TNBC cells previously^33^. The 3D TNBC cultures were exposed to Nos alone, DTX alone and Nos plus DTX, and the viabilities of both untreated and treated cultures were determined. Treatment with Nos plus DTX combination led to disintegration of 3D spheres of drug-resistant MDA-MB-231 TNBC cells when compared with their respective controls (Fig. 2A). Number of mammospheres of drug-resistant TNBC cells in each treatment group (Control, Nos alone DTX alone, Nos presensitization followed by DTX) was quantified microscopically (2B). The number of mammospheres decreased significantly (p < 0.01) in Nos plus DTX group compared to control. In terms of mammospheres number, there was no significant difference between control and Nos treatment groups, again confirmed the Nos alone treatment did not affect the cell viability. The cell viabilities of both wild type and drug-resistant TNBC cells were determined by alamar blue-based assay as shown in Fig. 2C. The marked reduction (p < 0.01) in cell viability of wild-type and drug-resistant TNBC cells was observed which were treated with Nos presensitization followed by DTX.

**Table 1:** The comparative cell viability of triple negative breast cancer cells 2D versus 3D.

Treatment Group	Wild type TNBC cells (% viability)	Resistant TNBC cells (% viability)
(**A**) Cell viability in 2-Dimensional culture (relative to untreated control)		
Noscapine	97	99
Docetaxel	61	74
Noscapine + Docetaxel	34	52
(**B**) Cell viability in 3-Dimensional culture (relative to untreated control)		
Noscapine	96	98
Docetaxel	73	82
Noscapine + Docetaxel	56	70

Viability of noscapine, docetaxel and combination of noscapine pre-sensitized followed by docetaxel treatment of MDA-MB-231 wild type and drug resistant MDA-MB-231 cells in 2D (Table 1A) and 3D (Table 1B) alginate scaffold system by alamarBlue.

We next found that there was a significant (p < 0.05) difference in the cell viability of 3D culture systems. Compared to conventional 2D systems, approximately 1.3 fold increase in the cell viability was observed in 3D models (Table 1A, and 1B). These results implicate that drug-resistant MDA-MB-231 breast cancer cells demonstrated higher cell viability than their MDA-MB-231 wild type cell counterparts.

### Oral administration of Noscapine in combination with intravenous docetaxel causes inhibition of wild type and drug resistant xenografted TNBC tumors

**Figure 3:**
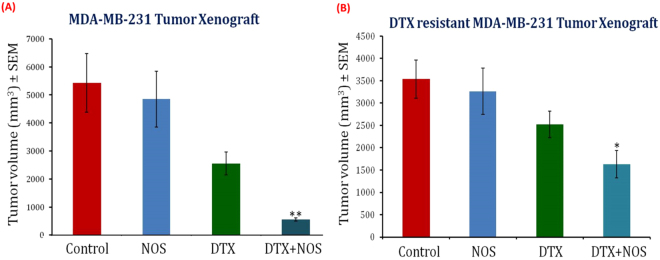
Noscapine oral administration followed by docetaxel liposomes significantly inhibits the tumor growth in wild type and drug resistant MDA-MB-231 breast tumor bearing nude mice. (**A**) Wild type MDA-MB-231 breast tumor xenografts. (**B**) Drug resistant MDA-MB-231 breast tumors xenografts tumor volume of each treatment group were collected at the end of the study. Breast tumor volume was significantly lower in the combination group as compared to other groups. Breast tumor volume data were given as mean ± SD (n = 6). *Indicates a significant (p < 0.01) difference compared with control. Error bars represents ± SD.

Further, we investigated the effects of Nos chemo-sensitization followed by DTX treatment on DTX resistant MDA-MB-231 orthotopic xenograft tumor bearing nude mice. The single-agent Nos sensitization in combination drug schedule were designed to reflect a clinically relevant approach with DTX (5 mg/kg body weight) administered intravenously twice a week and Nos (100 mg/kg body weight) fed by oral-gavage on a daily basis. At the end of the treatment, vehicle treated control mice (PBS) showed unrestricted tumor growth (Fig. 3A,B). Although single agent drug regimens decreased tumor growth and progression as compared to control (only PBS).

First, the *in vivo* antitumor efficacy of Nos, DTXL, or their combination was investigated in wild type MDA-MB-231 TNBC orthotropic xenograft tumor bearing nude mice as described in methods as per our previously published studies^32^. As shown in Fig. 3, the treatment groups showed significant (p < 0.001) tumor growth inhibition compared to the Nos only and control groups. Although oral administration of Nos followed by DTXL treatment resulted in reduced breast tumor volume, a significantly higher reduction in the tumor volumes were noted in the Nos plus DTXL group when compared with Nos alone or DTXL alone treated groups. Combination of Nos followed by DTXL group showed 5.49 and 3.25 fold reduction in tumor volume compared to Nos and DTXL group, respectively (Fig. 3A). In particular, the tumor volume and tumor size in the DTXL with Nos sensitization group showed a pronounced reduction compared with all other treatment groups (Fig. 3A), indicating a statistically improved antitumor effect of DTXL after Nos sensitization. In terms of body weight, Average body weight of treatment group (for DTX treated group 24 g ± 1.9 and for Nos plus DTX group 24 g ± 1.6) was higher than control group (22.5 g ± 1.2) indicating that the treatment has no apparent toxicities on body weight. Moreover, we observed that animals in control group were weaker than treatment group. Lower body weight and compromised movement could be attributed to solid tumor induced cachexia condition in control animals. Our *in vivo* studies revealed that DTXL with Nos sensitization exerts superior anticancer effects in wild type TNBC *in vivo* models.

Further, we extended our study to investigate the therapeutic efficacy of Nos pre-sensitization to overcome the DTXL resistance and *in vivo* studies were conducted in drug-resistant TNBC xenografts (Fig. 3B). The tumor growth inhibition was different in drug resistant MDA-MB-231 xenografts when compared to MDA-MB-231 wild type xenografts. Tumor growth of Nos pre-treated DTXL treatment showed significant (p < 0.05) reduction compared to DTXL alone. In drug-resistant xenografts, tumor volume was decreased 2.33 and 1.41 fold in xenografts treated with Nos followed by DTX liposomes significantly (p < 0.05) compared to Nos and DTXL alone respectively. Although, the reduction in tumor volume and tumor size was less when compared to wild type TNBC xenografts, these observations suggest that Nos pre-treatment overcomes the resistance of DTX efficiently in breast tumor xenografts. Administration of Nos as a chemo-sensitizer in conjunction with DTXL did not affect the body weight of the treated mice indicates safety of the DTX liposomes and Nos plus DTXL combination. These results suggesting that Nos treatment had no apparent cytotoxic side effects and the combined approach might be considered a potentially suitable strategy for treating TNBC.

### Noscapine chemo-sensitization overcome drug resistance by inhibiting the expression of multi-drug resistance proteins

**Figure 4:**
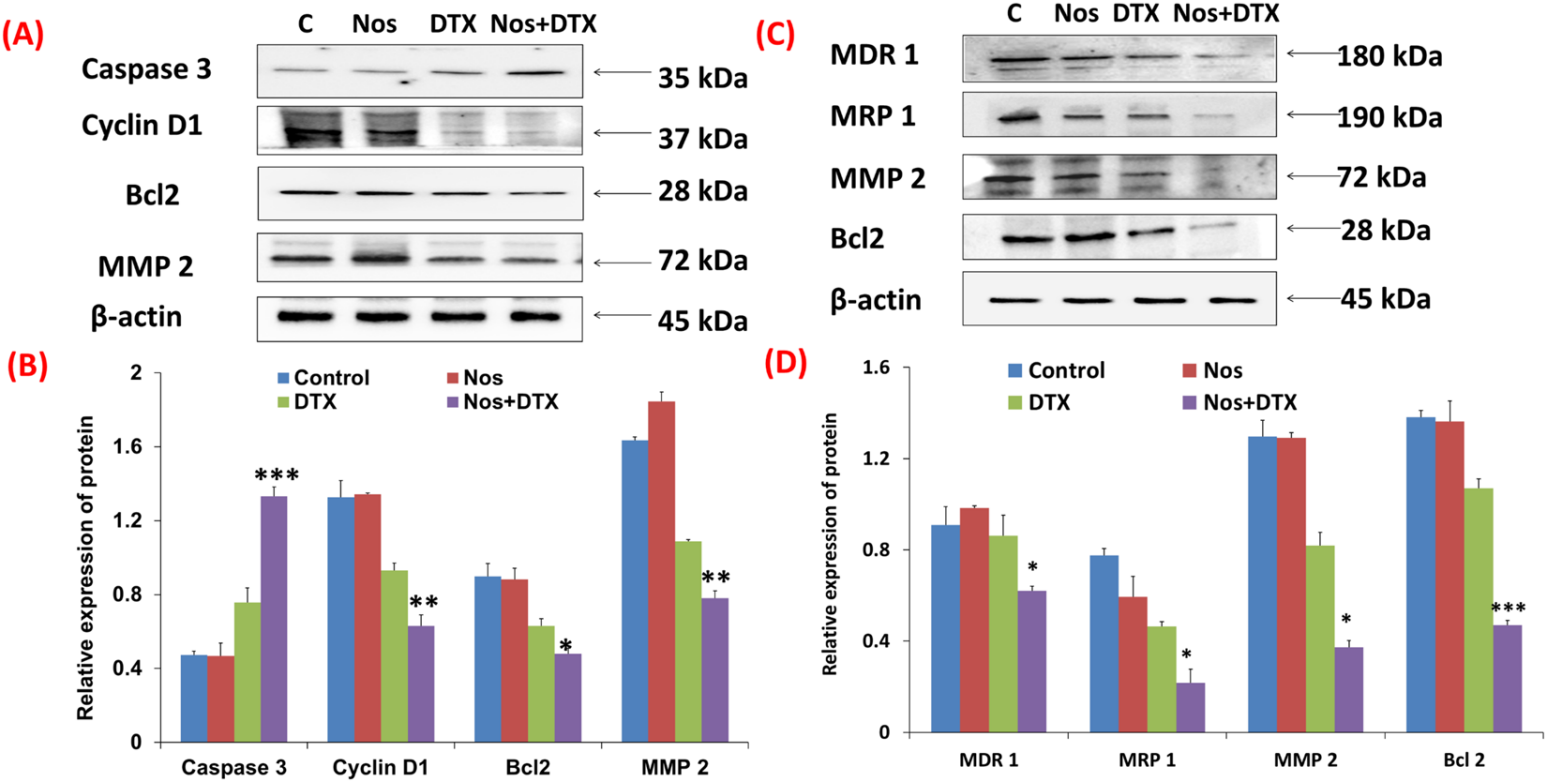
Western blot analysis of apoptotic and multidrug resistance proteins expression in wild type and drug-resistant triple negative breast cancer xenografts. (**A**) The protein expression of caspase 3, cyclin D1, bcl-2, MMP-2 and β-actin levels in wild type breast tumor xenografts of representative images were detected by western blot. (**B**) The intensity of indicated proteins was quantified by densitometric analysis and β-actin was used as a housekeeping protein. (**C**) Representative images of MDR 1, MRP 1, MMP-2, bcl-2 and β-actin in drug resistant TNBC tumor lysates of equal amounts, and (**D**). The intensity of indicated proteins expression in drug-resistant TNBC tumor lysates were quantified by densitometric analysis. Data are calculated from triplicate experiments and presented as mean, and error bars refer to SD. *Indicates a significant (**p < 0.01, ***p < 0.001) difference compared with control. Full-length blots were included in a Supplementary Information file as Figures S1 and S2.

We next studied the possible mechanism of Nos effect on cytotoxicity of DTX in wild type and DTX-resistant xenograft tumors. Since caspase 3, cyclin D1, bcl-2 and matrix metallo proteinase 2 (MMP-2) are key regulators in the cell cycle, apoptosis and extracellular matrix, we investigated the protein expression level in treated animal groups (Fig. 4) and full-length blots were included in a supplementary information file as Figures S1 and S2. Our previous *in vitro* study revealed that in wild type TNBC cells, Nos at low concentrations followed by DTX treatment inhibited growth of MDA-MB-231 cells in part by inducing apoptosis and stimulating activation of pro-apoptotic, stress-activated protein kinases (SAPKs), phosphorylated p38 and JNK1/2, Akt, bcl-2 and survivin^32^. In the present study, we found that the expression of apoptosis regulator bcl-2 was down-regulated (0.7 fold) significantly (p < 0.05) and caspase3 were upregulated (1.4 fold) markedly (p < 0.001) in Nos plus DTX combination compared to Nos alone and control groups (Fig. 4A and B). Cell cycle regulator cyclin D1 expression was also decreased (1.3 fold) significantly (p < 0.01) in Nos pre-sensitized animals (followed by DTX treatment) than other treatment groups. Our western blot analysis also showed that Nos pre-sensitization markedly (p < 0.001) inhibited the expression of MMP-2 (1.7 fold) in the combination group than other treatment groups.

To further verify the Nos pre-sensitization effect can overcome DTX resistance in DTX resistant xenograft tumors, we further analyzed the multidrug resistance proteins. Consistent with these findings, our western blot analyses of drug resistant TNBC tumor lysates in Fig. 4C and D show that Nos followed by DTX treatment inhibited the resistance marker MDR 1 (ABCB1) in drug-resistant TNBC cells. The expression of MDR 1was significantly (p < 0.05) down regulated (1.1 fold) in Nos pre-treatment followed by DTX treated cells when compared to control and DTX only treated cells (Fig. 4C and D). It is of note here that another resistance related protein MRP1 expression was also found to be higher in control lysates. On treatment with DTX after Nos chemo-sensitization, MRP 1 was significantly (p < 0.01) down regulated (1.13 and 1.9 fold) in DTX alone and DTX after Nos chemo-sensitization, respectively compared to untreated control xenografts (Fig. 4B). In consistent with the wild type TNBC *in vivo* data, ant-apoptotic protein bcl-2 (p < 0.001) and MMP-2 expression was also down-regulated significantly (p < 0.01) in combination group than other treatment groups. Thus, these findings highlight the potential of Nos pre-sensitization followed by DTX treatment and could be a clinically important combination to overcome the DTX resistance of breast cancer. Whether and to the extent such robust inhibition of drug resistant proteins by Nos pre-treatment followed by DTX treatment contributes to its superior TNBC growth inhibitory effects remain to be clarified.

### Decreased multidrug resistance related protein 1 (MRP1) expression in xenografted DTX resistant TNBC breast tumors

**Figure 5:**
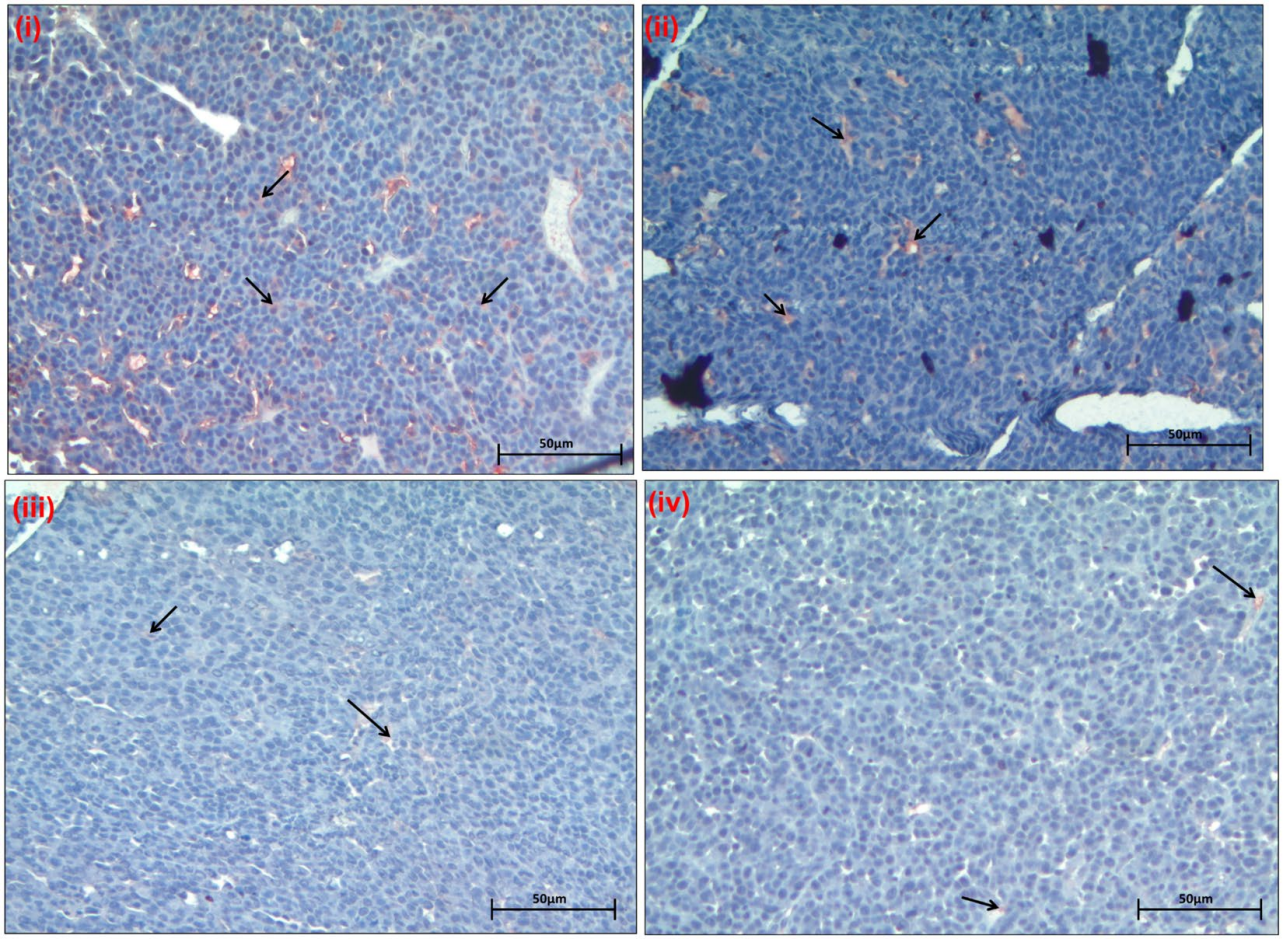
Immunohistochemical analysis of breast tumor section for multidrug resistance related protein 1 (MRP1). Animals were divided into four groups and treated as (i) Control, (ii) Noscapine only, (iii) Docetaxel only, (iv) Noscapine plus docetaxel. Noscapine at low concentration in conjunction with DTX treated groups showed marked reduction (brown color staining showing with arrows) in expression of drug resistant marker MRP1 than other treatment groups. Representative images shown at 200 × magnification (micron bar = 50 µm).

Further, the effect of Nos on the expression of MRP 1 in the nude mouse xenograft model was investigated by immunohistochemistry. The TNBC xenograft tumor sections revealed that the decreased staining for multidrug resistance protein MRP 1(shown with arrows in Fig. 5) in animals which were treated with oral administration of Nos followed by intravenous injection of DTX group. MRP 1 expression was markedly decreased as compared to animals which were treated with DTX alone. High amount of MRP 1 expression was found in control mice. The data shown in Fig. 5 collectively demonstrates that Nos pre-sensitization followed by DTX treatment overcome its drug-resistance.

## Discussion

Triple negative breast cancer (TNBC) has more aggressive disease progression with limited treatment options due to the lack of standard chemotherapy^34–36^. However, multidrug resistance (MDR) in cancer cells has remained as a significant obstacle in the achievement of efficient chemotherapy^37,38^. Due to multidrug resistance nature of tumor, use of nanocarriers like liposomal formulations may deliver their payloads of drugs more efficiently to cancer cells due to increased permeability and retention effect. Combining liposomal formulations along with natural compounds can improve the therapeutic efficacy of cytotoxic agents and possibly reverse MDR. To test this hypothesis, we investigated the therapeutic potential of Nos as a sensitizer at low concentrations in conjunction with DTX to overcome drug-resistant TNBCs. To our knowledge this study is the first attempt to identify a novel combination of Nos pre-sensitization at low concentrations in conjunction with DTX formulations demonstrating inhibition of growth of wild type and drug-resistant TNBC cells *in vitro* as well as *in vivo*.

DTX inhibited the growth of wild type as well as drug-resistant TNBC cells as shown in Fig. 1. In our previous study, we have used low dose 4 µM Nos and 0.4 µM DTX for *in vitro* cytotoxicity but in the present study we have used 8 µM Nos and 0.8 µM DTX. Usually, resistant cell line needs higher concentration of drug compared to wild-type cells, therefore, we have used 8 µM Nos and 0.8 µM DTX. The reason why we used different concentrations of Noscapine and DTX as compared to our previous study because we did not get IC50 (half maximal inhibitory concentration). Therefore, we have used higher concentration of Nos and DTX to get the IC50 in DTX resistant TNBC cells.

It is important to note here that killing of drug-resistant cells which were treated with DTX was more significant in Nos pre-sensitized cells than without Nos pre-treatment suggesting that TNBC growth inhibition by microtubule class of compounds can be used to overcome the drug-resistance of TNBC. This is further supported by our mammosphere studies where Nos sensitization and DTX was effective in disrupting mammospheres of wild type as well as drug-resistant TNBC cells. In mammalian tissues and cells connect not only to each other, but also to support structures called extracellular matrix (ECM). The cells grow within an organized three dimensional (3D) matrix and their behavior is dependent upon interactions with immediate neighbors and the ECM^39^. We have utilized Algimatrix 3D platform to culture both wild type and drug resistant TNBC cells because 3D cell culture models create a pragmatic microenvironment and mimic *in vivo* systems, which helps to understand cell-cell interactions^40,41^. In the current study, Nos at low concentrations in conjunction with DTX combination was more effective to disintegrate mammospheres than either agent alone. Our laboratory has previously demonstrated that 3D cell culture scaffolds (AlgiMatrix^TM^) serve as a valid platform for the development of more physiologically relevant culture systems for cancer biology^33,42^. Collectively, our current *in vitro* 2D and 3D studies demonstrate that this combination has unique ability to target resistant cells to suppress growth of drug-resistant TNBC cells. Zhou *et al*. showed that noscapine binds to tubulin at a different site than paclitaxel and causes mitotic arrest in paclitaxel-resistant ovarian carcinoma cells^14^. It has been demonstrated that the intracellular mediators in 3D multicellular morphologies showed greater resistance to chemotherapy than in monolayers in three different endometrial cancer cells such as Ishikawa, RL95-2, and KLE cell lines^43^. It was shown that doxorubicin had less effect on proliferation and induced less apoptosis in 3D multicellular structures of high grade cancer cells (RL95-2 and KLE cell lines) than in cell monolayers. These observations have important implications with regard to the *in vitro* study of anticancer treatments.

Liposomal formulation of DTX was developed to improve the solubility of DTX and its long circulation and sustained release of DTX. Nanosized liposomes are reported to selectively accumulate in solid tumor due to EPR effect. However, deeper penetration in tumor tissue is severely restricted by collagen rich tumor stroma and other components of tumor ECM. Administration of Nos orally at low concentrations disrupts the extracellular matrix network due to its anti-fibrotic activity, therefore, DTX liposomes were more permeable to TNBC tumors^32^. Nos has been reported to have *in vitro* anticancer activity for a wide variety of cancers and administration of Nos does not have toxic side effects on any other organs *in vivo* ^15^. In the doses of 100 mg/kg, Nos after oral administration lead to the sensitization of tumor, so DTX liposomes reach tumor more efficiently and reduce the tumor volume of TNBC xenografts. These findings are in agreement with Shen *et al*. (2015) who conducted a study investigating the cytotoxic effects of the combination of noscapine and cisplatin in cisplatin-resistant human ovarian cancer SKOV3 cells *in vitro* and *in vivo* null mouse xenograft model. Noscapine inhibited proliferation of the SKOV3 and SKOV3/cisplatin-resistant ovarian cancer cells dose-dependently^44^.

Our recently published *in vitro* data suggests that the treatment of wild type TNBC cells with Nos at low concentrations stimulated activation of stress-activated kinases p38 and JNK1/2 in a time and dose-dependent manner^32^. Hence, we construe from this study that lower concentrations of Nos act as a chemo-sensitizer and treatment with DTX may produce superior anticancer effect that warrants further investigation for its potential clinical applications. To extend to our previous study, we extrapolated present study to molecular level *in vivo* wild-type and drug-resistant xenografts to investigate the antitumor efficacy of Nos chemo-sensitization to overcome the drug resistance. Decreased expression of bcl-2, cyclin D1 and MMP 2 with combination treatment in wild type TNBC tumors is in agreement with our previously published studies which demonstrated that Nos also downregulates the expression of various cell cycle regulators and survival proteins^14,15,18,32^. Increased expression of pro-apoptotic factor caspase 3 in wild type xenograft breast tumors, correlates with the work of Shen *et al*., (2015) who showed that Nos increases the anti-cancer activity of cisplatin in ovarian cancer cells SKOV3/DDP by modulating the cell cycle and activating apoptotic pathways^44^.

**Figure 6:**
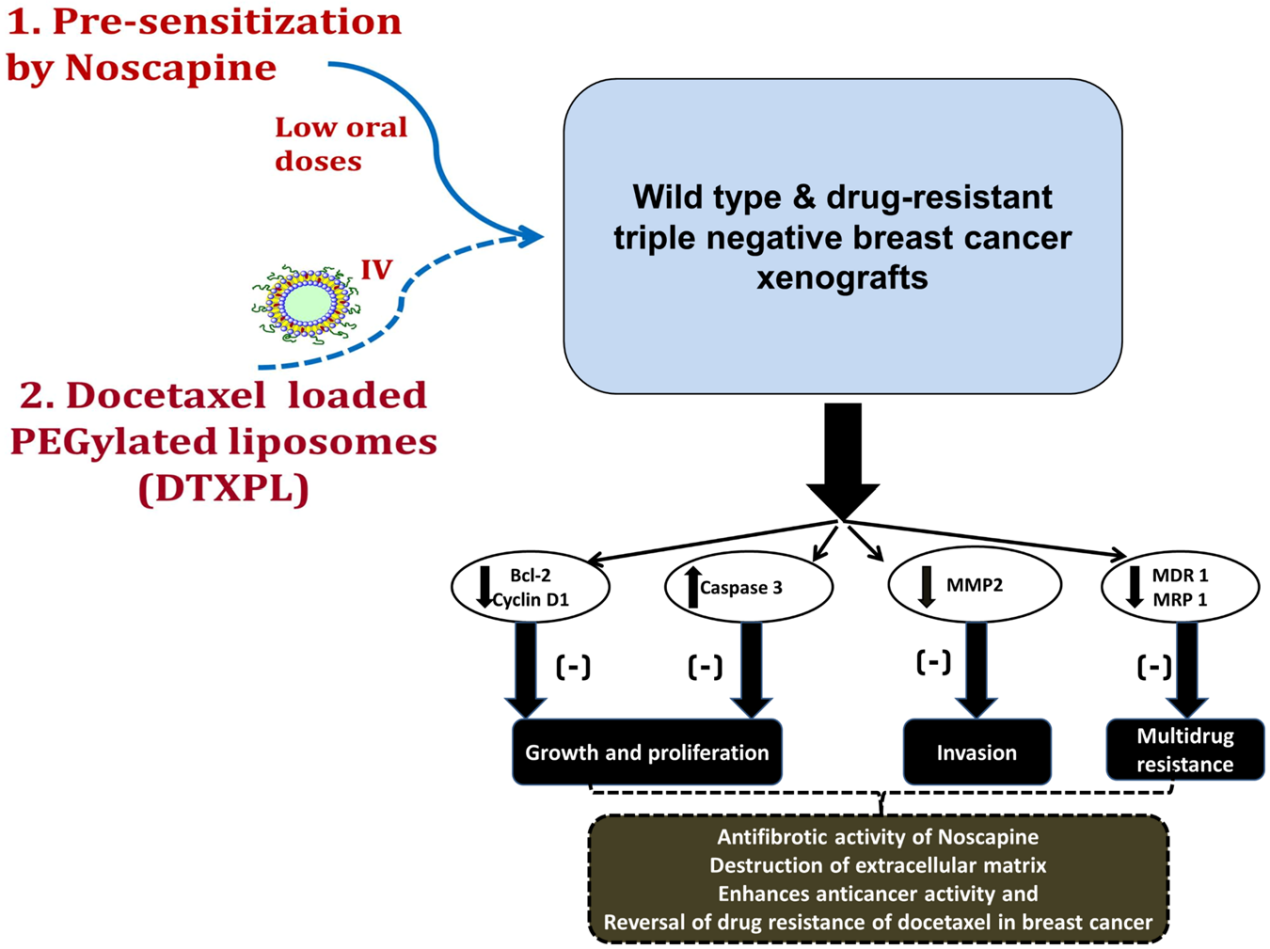
Illustrated a combination of noscapine pre-sensitization at low concentrations followed by docetaxel inhibits growth of wild type and drug-resistant TNBC cells. Noscapine oral administration enhanced the anticancer activity of docetaxel in drug resistant triple negative breast cancer by augmenting the tumor bioavailability of docetaxel liposomes and chemo-sensitizing the tumor to docetaxel. The combination of noscapine plus docetaxel liposomes administration down-regulated the expression of anti-apoptotic factors bcl-2, cyclin D1 and multidrug resistance proteins such as MDR 1, MRP 1 as well as extracellular matrix metalo protein 2 (MMP-2) and augmented levels of pro-apoptotic caspase-3 expression. These findings provided a promising strategy to overcome multidrug resistance by combined delivery of noscapine pre-sensitization followed by docetaxel anticancer agents to target tumor cells more effectively.

ATP-binding cassette (ABC) transporters such as MDR1, MRP1 and BCRP (family of multidrug resistance proteins) play a crucial role in mediating drug resistance in cancer cells^45–47^. Our current studies further revealed that drug-resistant TNBC cells have decreased expression of key regulators of resistance such as MDR1, MRP 1 and anti-apoptotic protein bcl-2 in drug-resistant xenograft breast tumors (Fig. 4). These results indicate that Nos increases the sensitivity of xenografts to DTX, which led to increased apoptosis and decrease resistance of TNBC tumors and correlates with more aggressive phenotype of tumor and progression of disease^48–50^. Decreased expression of bcl-2 in drug resistant tumors suggests that it may be involved in the resistance mechanism in these TNBC cell lines. Our results showed that Nos significantly suppressed the invasive ability of MDA-MB-231 cells in xenografts in parallel with down-regulation of MMP2. Nos mediated disruption of tumor ECM will enhance the tumor penetration and tumor bioavailability of DTX liposomes corroborated well with previous studies^51,52^. Immunohistochemical expression of MRP 1 positive staining were detected and the intensity of MRP 1 staining was less in the combination than other treatment groups, agreement with the previous studies^53–55^. Su *et al*. (2011) also found that noscapine sensitizes cisplatin-resistant ovarian cancer cells by inhibiting hypoxia inducible factor-1 alpha (HIF-1α)^56^. Although, these results need further evaluation, the present findings do support the conception that Nos may offer a novel therapeutic strategy for drug resistant TNBCs (Fig. 6).

In conclusion, to our knowledge this study is the first attempt to identify a combination of Nos pre-sensitization at low concentrations in conjunction with DTX inhibits growth of wild type and drug-resistant TNBC cells illustrated *in vitro* as well as *in vivo* (Fig. 6). Nos oral administration enhanced the anticancer activity of DTX in drug resistant TNBC by enhancing the tumor bioavailability of DTX liposomes and chemo-sensitizing the tumor to DTX. This study provides a basis for improving efficacy of chemotherapy effect on drug resistant TNBCs and sheds light on new insights to the development of novel clinical therapeutics.

## Materials and Methods

### Chemicals and Reagents

Noscapine, docetaxel were purchased from Sigma Chemicals, St. Louis, MO, USA and AK Scientific Chemicals, CA, USA. The TNBC cell lines MDA-MB-231 cells were obtained from American Type Culture Collection (Rockville, MD, USA). Cells were grown in DMEM:F12K medium (Sigma, St. Louis, MO, USA) supplemented with 10% fetal bovine serum and antibiotic solution purchased from life technologies, USA. The cells were maintained at 37 °C in the presence of 5% CO_2_. All other chemicals were either reagent or tissue culture grade.

### Generation of drug-resistant TNBC cells

Human MDA-MB-231 TNBC cells were cultured in the chronic presence (>6months) of DTX. The wild-type cells were initially treated with 200 nM DTX for 2–3 weeks, followed by escalation to 400 nM, 1 µM, and 2 µM doses over a period of 3–4 weeks for each dose till resistance developed and the cells became well adapted to growth in 1 µM dose of DTX for their routine culture. Subsequent, routine maintenance of the resistant cells in the presence of the respective drug was continued and resistant subline for the TNBC cells were characterized for their inhibitory concentration (IC)50 of respective therapeutic by the crystal violet-based cytotoxicity assays.

### Cytotoxicity of docetaxel resistant cells after Noscapine chemo-sensitization

*In vitro* inhibition of cell growth was assessed in Nos on DTX resistant TNBC cells by crystal violet cytotoxicity assay. The wild type and DTX resistant MDA-MB-231TNBC cells were plated in 96-well micro titer plates, at a density of 1 × 10^4^ cells/well and allowed to incubate overnight and were treated with various dilutions of Nos made in cell growth medium (10 to 160 µM) from Nos stock solution in DMSO. To study the interaction between Nos and DTX, the treatment strategy included as cells treated with (i) control (ii) only Nos (8 µM) for 24 h (iii) only DTX (0.8 µM) (iv) Nos 8 µM for 24 h followed by DTX 0.8 µM for 24 h. In group (ii) and (iv), Nos was discarded after 24 h and replaced with fresh media and DTX, respectively and viability was assessed by crystal violet assay^32^. The absorbance was measured by a microtiter plate reader (Spectramax 190, Molecular devices, USA) at 540 nm.

### Three-dimensional mammosphere assays in alginate scaffold 3D Breast Tumor Model

Initially, DTX resistant cells were pre-treated with Nos for 24 h then followed by DTX treatment. DTX (0.8 µM) was used to treat 3D alginate scaffolds seeded with 0.15 million cells on 7, 9, 11 days post tumor cell seeding based on our previously published study^33^. Similarly in 96 well plates after seeding 15,000 cells per well, spheroids were treated with DTX (0.8 µM) on 7, 9, 11 days post cell seeding. The alamarBlue® assay was performed to determine number of cells at the end point. Results were compared with 2D culture systems.

### AlamarBlue® Assay

At 14 day in culture cell viability and metabolic activity was measured using the alamarBlue® assay which is based on the conversion of a non-fluorescent dye to the red fluorescent dye resorufin in response to chemical reduction of growth medium resulting from cell growth. Briefly, 10% alamarBlue® dye with respect to the volume of the medium in each well was added. After one hour of incubation, plates were read for fluorescence intensity at 530 nm & 590 nm wavelength for excitation and emission, respectively.

### Preparation and characterization of liposomes containing DTX (DTXL)

Docetaxel loaded PEGylated Liposomes (DTXPL) of 105.7 ± 3.8 nm particle size was prepared using modified hydration method as described in our previous report^27^.

### Establishment of TNBC cell-derived xenografts in immunocompromised mice

The experiments involving generation of DTX-resistant TNBC cell-derived sub-cutaneous xenografts were performed according to our previously published methods and protocols^23,32^ approved by the Institutional Laboratory Animal care & Use Committees at the Florida A&M University and all methods were performed in accordance with the relevant guidelines and regulations. Female, 5-weeks Balb/c nude mice were purchased from Charles River Laboratories (Horsham, PA). The orthotopic TNBC xenograft studies were carried out in female Balb/c Nude Mice. Following suitable acclimation of animals, 1.5 × 10^6^ wild-type and 1.0 × 10^6^ drug-resistant MDA-MB-231 TNBC cells were re-suspended in 100 µl of phosphate buffer solution (PBS), and implanted in the mammary fat pads using a 27-gauge needle Tumors were allowed to grow unperturbed for 10–14 days. When tumors became palpable, the mice were randomly assigned to treatment or control groups of six animals each. Mice were treated with control, PBS only, Nos (100 mg/kg), DTXL (5 mg/kg), or Nos plus DTXL. Nos was administered by oral gavage every alternate day for 2 weeks while DTXL was given by intravenous route weekly twice by tail vein. For present study, we have monitored animals every alternate day after the last dose of DTX up to 4 weeks. Study was terminated when more than 50% of control animals were unable to move around due to large tumor. All the animals were euthanized using carbon dioxide. Body weight and tumor volume was measured for assessment of therapeutic efficacy Nos and DTX. Tumor volumes were calculated by the modified ellipsoidal formula. Tumor volume = 1/2(length × width^2^). Representative tumor samples were stored at −80 °C for subsequent analysis.

### Western blot assays

Total proteins were extracted using cell lysis buffer (1% NP-40, 150 mM NaCl, 50 mM Tris pH 7.4, 2 mM EDTA, and protease inhibitor cocktail). Cell lysates were subjected into 7.5% or 10% SDS-PAGE. Separated proteins were transferred onto nitrocellulose membrane (Bio-Rad). The transferred membranes were blocked with 5% skim milk in Tris-buffered saline containing 0.05% Tween-20 (TBS-T) for 1 h at room temperature, and then incubated overnight with primary antibodies MDR1 (ABCB1), MRP 1(ABCC1), cyclin D1, bcl-2, MMP-2 and β-actin diluted at 1:1000 in 5% skim milk in TBS-T at 4 °C and followed by secondary. Finally, protein levels were visualized using ChemiDoc XRS + Imaging system (Bio-Rad, USA).

### Statistical analysis

Statistical analysis was performed using unpaired Student’s t-test. A p-value less than 0.05 between treatment groups was considered significantly (*p < 0.05) different from untreated controls.

## Electronic supplementary material

Supplementary Figure S1: Full length blots of Figure 4.
