# Supplementary material for: Reversal of drug-resistance by noscapine chemo-sensitization in docetaxel resistant triple negative breast cancer
Source: Sci Rep. 2017 Nov 20;7:15824. doi: 10.1038/s41598-017-15531-1 (PMC5696458; doi:10.1038/s41598-017-15531-1)

Reversal of drug-resistance by noscapine chemo-sensitization in docetaxel resistant triple negative breast cancer

Ravi Doddapaneni[1, 2], Ketan Patel[1,3], Nusrat Chowdhury[1], Mandip Singh[1#]

1. College of Pharmacy and Pharmaceutical Sciences, Florida A&M University, Tallahassee, FL 32307, USA.
2. Department of Ophthalmology, Bascom Palmer Eye Institute, University of Miami Miller School of Medicine, Miami, FL 33136, USA.
3. College of Pharmacy and Health Sciences, St. John's University, Queens, NY 11439, USA.

# Corresponding author

Mandip Singh, College of Pharmacy and Pharmaceutical Sciences, Florida A&M University, Tallahassee, FL 32307; Tel: (850) 561-2790; Fax: (850) 599-3813; E-mail: mandip.sachdeva@famu.edu

Supplementary Figure S1: Full length blots of Figure 4A. Immunoblotting analyses were performed to confirm the protein expression of the caspase 3, Cyclin D1, bcl2 and MMP 2 in wild-type tumor lysates of TNBC tumor xenografts.

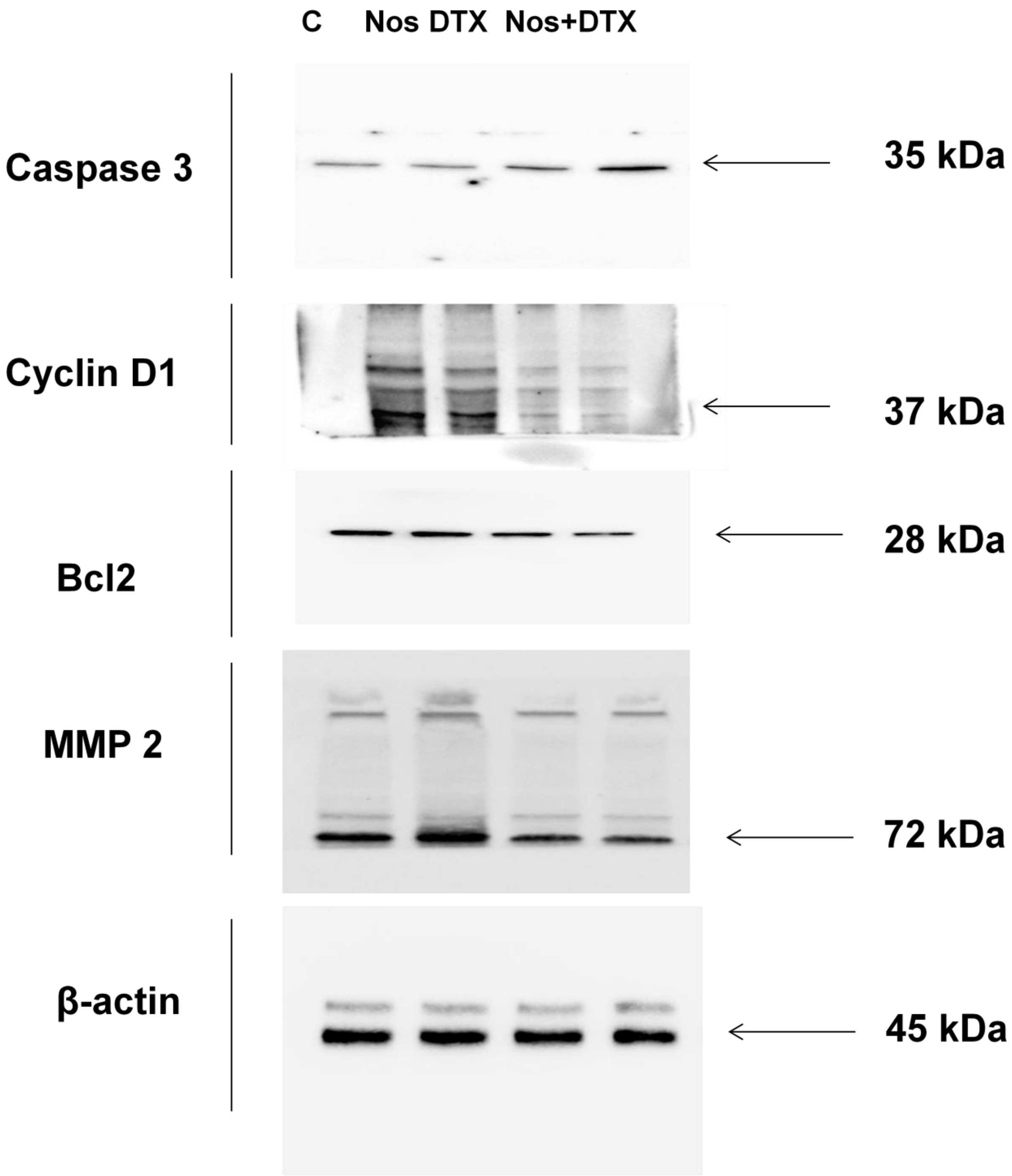

Supplementary Figure S2: Full length blots of Figure 4C. Immunoblotting analyses were performed to confirm the protein expression of the MDR 1, MRP 1, MMP 2 and bcl2 in drug-resistant tumor lysates of TNBC tumor xenografts.

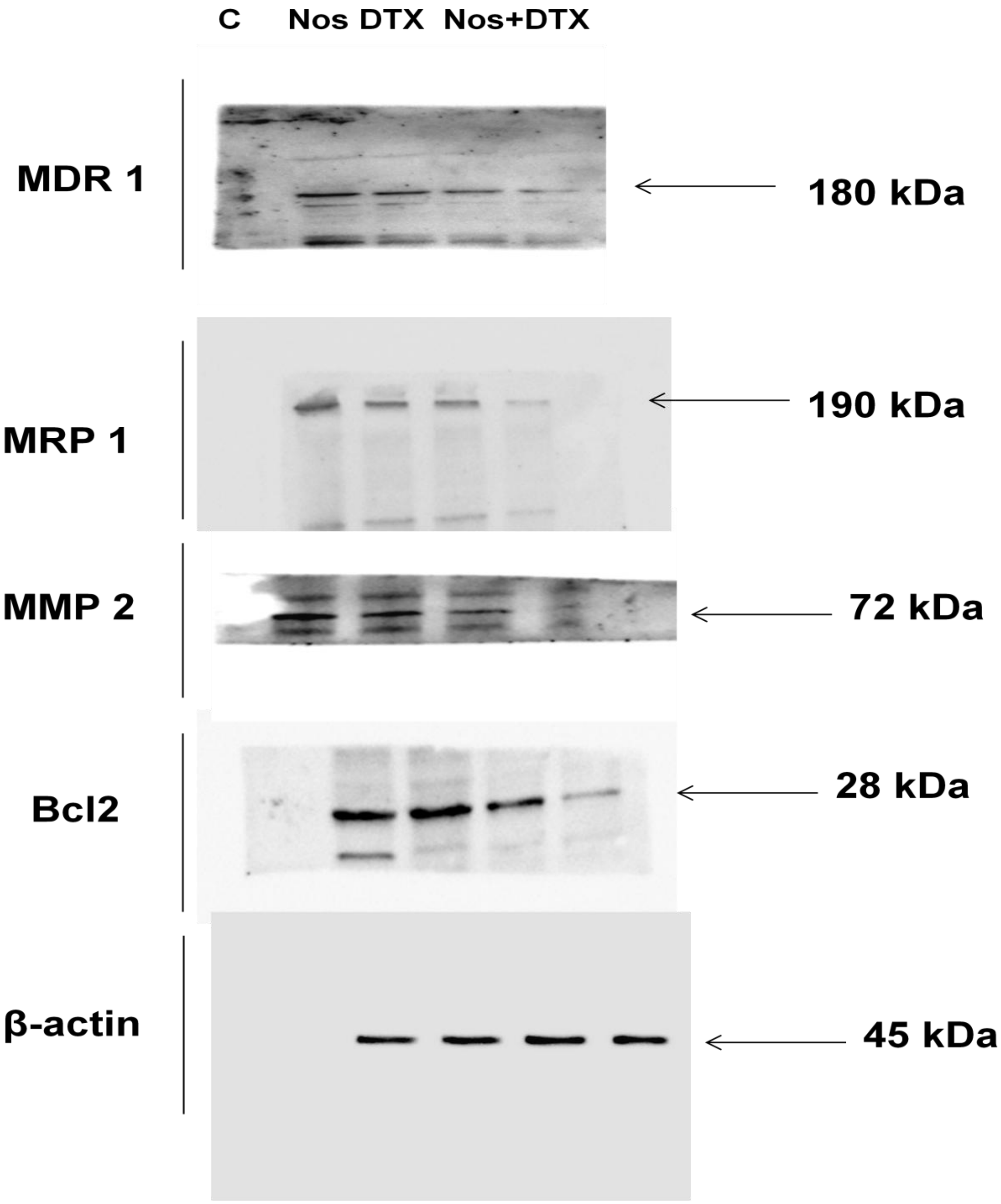

Supplement: Supplementary file 1 — Supplementary Figure S1: Full length blots of Figure 4. [file 41598_2017_15531_MOESM1_ESM.pdf]
